# Reliability of lumbar spinal palpation, range of motion, and determination of position

**DOI:** 10.1186/1471-2474-8-103

**Published:** 2007-10-30

**Authors:** Michael Troke, Dale Schuit, Cheryl M Petersen

**Affiliations:** 1 York St. John University, York, UK; 2 University of Brighton, Brighton, UK; 3 Department of Physical Therapy, Governors State University, University Park, Illinois, USA; 4 Department of Physical Therapy, Rosalind Franklin University of Medicine and Science, 3333 Green Bay Road, North Chicago, Illinois, USA; 5 Division of Human Services, Programs in Physical Therapy, Concordia University Wisconsin, Mequon, Wisconsin, USA; 6 Northwestern University, Department of Physical Therapy and Human Movement Sciences, 645 North Michigan Avenue, Suite 1100, Chicago, Illinois, USA

## Abstract

**Background:**

The study purposes were to investigate the level of agreement of palpation of lumbar spinous processes between examiners, test-retest repeatability of lumbar spine range of motion, and the reliability of upright position measures in asymptomatic subjects.

**Methods:**

The modified CA 6000 spinal motion apparatus with a new skin fixation system was used by three operators for the test-retest spine measurements (3 days apart), and to obtain measures at one session of spinal position. Mean ranges of motion in all planes for 22 asymptomatic subjects were reported using the Intra-class Correlation Coefficient.

**Results:**

Overall, differences in palpation agreement for lumbar segments occurred in three subjects and did not affect range of motion values. For upright spinal position, ICC (2,3) values for sagittal, coronal, and horizontal plane positions were 0.96, 0.80, and 0.98 respectively. There were statistically significant differences between examiners for position values, determined by the Bonferroni t-test (p < 0.05), but the magnitude of the differences were 2 degrees or less, and not considered clinically important.

**Conclusion:**

Results suggest that lumbar spinal motion measurements and position determination between different operators can be consistent particularly if utilizing the modified instrument. Static lumbar position also appears to be recorded reliably between different operators. Results justify progression to multi-center lumbar research using the modified CA 6000 and the work is considered relevant to medical clinicians working with spinal dysfunction, surgical interventions, or occupational health.

## Background

Previous work on the development of a new form of skin fixation for use with the CA 6000 has been fully described [1]. The reliability and repeatability of the CA 6000 measures produced with the new fixation system have also been reported for the lumbar and thoracic spinal regions [2,3]. Validity of measures was reported [4] and a normative database for lumbar spinal motion was introduced [5] and subsequently published in full [6]. The development of the skin fixation system for the CA 6000 has provided clinicians and researchers with the opportunity to measure spinal movement reliably and validly with 6 degrees of freedom. Inter-rater reliability using multiple investigators performing individual palpation with skin fixation has not yet been assessed with the modified instrument. The previous intra/inter-rater reliability work [2,3], which involved standardized palpation by one examiner, was focused on establishing the instrument's repeatability when operated by different clinicians.

Recruitment of large numbers of subjects in a timely manner at multiple sites can be problematic in research. This present study, using the new skin fixation system with the CA 6000 instrument and 3 separate operators performing independent spinal palpation, was considered a potentially useful precursor to a multi-center approach towards research in spinal dysfunction.

The purposes of this study were: 1) to investigate the level of agreement of lumbar spine palpation between 3 separate examiners; 2) to determine the repeatability of measures of lumbar spinal range of motion by 3 examiners over 3 days; and 3) to determine the reliability of measures of upright lumbar position by 3 examiners.

## Methods

Twenty-two volunteer subjects were recruited (9 female and 13 male) to participate. Age, height and weight ranges, means and standard deviations were as follows: age range 22–38 yrs, mean 26.73 yrs, SD 4.58; height range 1.54–1.89 m, mean 1.73 m, SD 0.094; and weight range 56.25–107.5 kg, mean 74.06 kg, SD 14.66. All subjects were asymptomatic at the time of testing, which was defined as follows: no history of low back pain within the last 3 months; no history of recent trauma to the lumbar spine; no history of pathology of the lumbar spine (e.g. scoliosis, spondylolisthesis, herniated disc, degenerative joint disease). All subjects read and signed an informed consent form approved by the Institutional Review Board at Rosalind Franklin University. All three of the operators were experienced with the instrument from previous research. Two of the operators, B and C (CP and DS, respectively) were inexperienced in the use of the new skin fixation system developed by the third operator, A (MT). Operators B and C were trained by operator A prior to data collection. Each operator instructed, palpated and measured each subject separately and independently. A randomly repeated order of the 3 operators was used throughout the study. All subjects had read and signed informed consent forms.

On the first measurement day, each subject was asked to assume a side lying position on a plinth with their back facing away from the operator. The lumbar spine was palpated by the first operator to locate the L4–5 interspace, marked and cross-checked against the level of the uppermost iliac crest. The spinous process of S2 was identified followed by the spinous process of T12 which was palpated cephalad to the T12–L1 interspace and marked with a specific color by each operator. Palpation of the twelfth rib was also used to recheck the position of the T12 spinous process. Operators were not influenced by the marks of the other operators because the dorsal surface of the lumbar area of each of the subjects faced away from the operator during initial palpation and identification of vertebral levels. Once marked with the subject facing away in side lying, operators were not permitted to amend their vertebral level markings.

**Figure 1:**
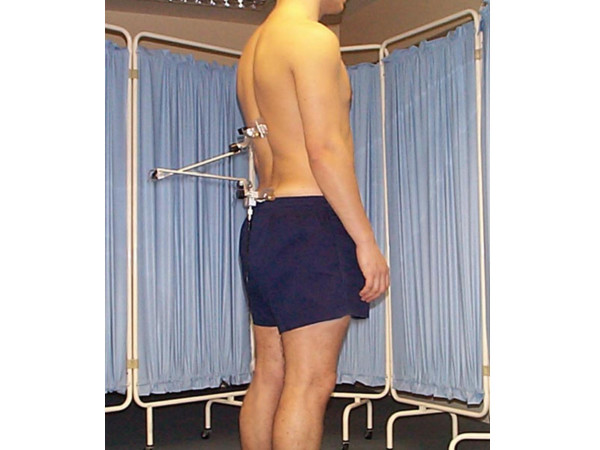
Subject with new skin pad fixation system and OSI CA 6000 Spine Motion Analyzer linkage.

Each subject then adopted approximately 50% of their flexion range of motion to provide a degree of skin tension over the marks. The operator applied the fixation pads over their own T12 and S2 markings. The UK manufactured skin fixation system was brought to the US for the purposes of this study (see Figure 1). Each operator was able to see the previous operator's marks in standing. At this point in the study, the palpation markings had not been compared or validated and so each operator was instructed to ignore those markings and to place the fixation pads only over their own markings. Each subject then stood facing the wall in their neutral standing posture. A marker was applied to the same wall directly in front of each subject, adjusted to each subject's eye height level to provide a visual target to assist the subject in determining the starting position for the range of motion measures.

The CA 6000 instrument was interfaced with a standard PC utilizing CA 6000 software (Orthopedic Systems, Inc., 30031 Ahern Avenue, Union City, CA 94587). The instrument linkage was secured onto the pad hooks. Each subject was instructed separately by each individual operator, (a randomly repeated order of the 3 operators was used throughout the study for each subject), and initially subjects were asked to carry out five familiarization/warm-up movements into full flexion then full extension, returning to the standardized start position. The instrument settings were zeroed on the computer. Each subject was asked to move on command into maximum lumbar flexion and extension. The movements were repeated 3 times and the data were then saved.

Each subject was asked to resume the start position, and to carry out five familiarization/warm-up movements, maximally into right lateral flexion, then maximally into left lateral flexion, returning to the start position. The instrument settings were zeroed on the computer. Each subject was asked to move on command into maximum lumbar right and left lateral flexion. The movements were repeated 3 times and the data saved.

Each subject resumed the start position and was asked to cross their arms placing their hands on the contra-lateral shoulder. Five familiarization/warm-up movements were carried out into full right axial rotation, and full left axial rotation, returning to the start position. Each subject was instructed to lead the rotation with their head, maintaining a horizontal arc of vision, rotating the thoracic and cervical spines at will in order to achieve maximum lumbar axial rotation. The instrument settings were zeroed on the computer. Each subject was then asked to move on command into maximum lumbar right and left rotation. The movements were repeated 3 times and the data was saved.

Each subject was then asked to assume their neutral standing posture. After verbal confirmation that they were indeed in their neutral standing posture, the CA-6000 instrument settings were zeroed on the computer. While standing quietly in the upright position, a baseline measurement was taken for the position of the lumbar spine in each of the 3 cardinal planes. Each subject was then asked to perform complete maximum motion of the lumbar spine once in each cardinal plane, and return to their neutral standing posture. A second upright position reading was taken. Each subject then repeated the same motions, and a third upright position reading was taken. The 3 upright position readings for each subject were saved. This entire procedure was then repeated for each subject by each of the two remaining examiners independently.

On completion of all measurements by all three operators on the first day, the pen marks were reinforced if required and surgical tape was applied over the marks to preserve them for the next session some days later. Palpation was not therefore repeated on the two subsequent testing sessions. Each subject was scheduled as was practicable at the same time each day for two additional testing sessions to minimize diurnal effects. For the remainder of the study, the complete set of motion measurements in flexion/extension, lateral flexion, and axial rotation were randomly repeated by each operator independently.

All range of motion data were subsequently collated and statistically analyzed using SPSS (SPSS Inc., 233 South Wacker Drive, 11^th ^Floor, Chicago, IL 60606) 8.0 statistical computer software package. Analyses of variance were calculated from the individual data across each subject and for each operator. Two-way analysis of variance was utilized, leading to the calculation of ICCs, type 2,3 for inter-operator and type 3,3 for intra-operator [7,8]. The results were analyzed separately in order to establish the reliability and repeatability for all the operators independently, and then combined. Static lumbar position measures were recorded as components of the upright posture in the 3 cardinal planes, with ANOVA determination using the GBStat program (version 6.5). ICCs type 2,3 were calculated [8]. Standard error of measurement (SEM) and variable error were also calculated for each subject. Variable error reflects the variability in subject performance around their mean response [9].

## Results

Palpation results for 19 of the 22 subjects (86.4%) showed substantial agreement, with no observable differences (complete overlap) of the blinded markings for the T12 and S2 vertebral levels. Data from three subjects (13.6%) indicated palpation differences for the T12 spinous process position. Differences of 2, 5, and 18 mm were found for the three subjects, respectively. These differences were between 2 operators for three male subjects.

Separate Excel spreadsheets were compiled for each operator's range of motion results. Mean ranges of motion and standard deviations were calculated for each of the primary motions and are summarized in Table 1. Comparison with the asymptomatic male and female age-related normative database [6] suggested that all subjects achieved ranges of motion which might be expected of 80% of the age-related general population. The difference in mean measured ROM between all three operators for all primary motions was found to be less than 1 degree on all occasions.

**Table 1:** Lumbar spinal mean ranges of motion by operator over all 3 days

Operator	Movement	Mean ROM (degrees)	Confidence Interval
A	Sagittal	89.9	(89.6, 90.2)
	Coronal	54.0	(53.7, 54.3)
	Horizontal	14.4	(14.2, 14.6)
			
B	Sagittal	90.6	(90.2, 91.0)
	Coronal	54.3	(53.7, 54.9)
	Horizontal	14.8	(14.5, 15.1)
			
C	Sagittal	90.5	(90.0, 91.0)
	Coronal	54.9	(54.5, 55.3)
	Horizontal	14.4	(14.1, 14.7)

Means and standard deviations of ROM values were calculated for all 3 operators across all three days for all subjects. These values are summarized in Table 2. Intra-operator ICC (3,3) were calculated for the 3 operators and are summarized in Table 3 and inter-operator ICC (2,3) are summarized in Table 4. A reliability coefficient of between 0.41 and 0.60 is characterized as 'moderate', between 0.61 and 0.80 is characterized as 'substantial' and an ICC of 0.81 or above as 'almost perfect [7].' All but one of the ICCs obtained from this study were either in the 'substantial' or 'almost perfect' categories.

**Table 2:** Lumbar spinal mean ranges of motion for all subjects measured by all operators on all days

Movement	Mean ROM (degrees)	Confidence Interval
Sagittal	90.3	(89.9, 90.7)
Coronal	54.4	(53.9, 54.9)
Horizontal	14.6	(14.3, 14.9)

**Table 3:** Intra-operator Intra-class Correlation Coefficients by operator over all 3 days

Operator	Movement	ICC (3,3)
A	Sagittal (Flex/Ext)	0.81/0.94
	Coronal (R/L)	0.86/0.95
	Horizontal (R/L)	0.86/0.63
		
B	Sagittal (Flex/Ext)	0.98/0.86
	Coronal (R/L)	0.81/0.57
	Horizontal (R/L)	0.83/0.71
		
C	Sagittal (Flex/Ext)	0.99/0.80
	Coronal (R/L)	0.87/0.78
	Horizontal (R/L)	0.72/0.92

**Table 4:** Inter-operator Intra-class Correlation Coefficients for all operators over all 3 days

Movement	ICC (2,3)
Sagittal (Flex/Ext)	0.82/0.73
Coronal (R/L)	0.71/0.82
Horizontal (R/L)	0.71/0.76

Values were recorded for the position of the lumbar spine in each of the 3 cardinal planes. Mean values were calculated for each operator and are summarized in Table 5. A negative value in the coronal or horizontal plane indicates a position to the left of midline. A positive value indicates a position to the right of midline. Standard error of mean (SEM) values are recorded in Table 6, and values for variable error are recorded in Table 7. Mean variable error values were all less than 2.5 degrees. ICC (2,3) for sagittal, coronal and horizontal plane positions were 0.96, 0.80, and 0.98, respectively. Statistically significant differences between examiners for various position values were determined by the Bonferroni t-test (p < 0.05). The actual magnitudes of the differences were approximately 2 degrees or less, which the authors considered to be clinically non-significant.

**Table 5:** Mean lumbar position values (sd) for all trials for all subjects by operator

Operator	Sagittal* (degrees)	Coronal** (degrees)	Horizontal*** (degrees)
A	24.8 (7.2)	0.9 (2.8)	-2.3 (4.4)
B	25.4 (8.0)	3.3 (2.2)	-1.2 (3.8)
C	26.8 (7.6)	1.2 (3.9)	-2.8 (4.1)

* Positive value indicates position of extension; negative value indicates position of flexion.

** Positive value indicates position of right lateral flexion; negative value indicates position of left lateral flexion.

*** Positive value indicates position of right axial rotation; negative value indicates position of left axial rotation.

**Table 6:** Lumbar position standard error of measurement for all trials for all subjects by operator

Operator	Sagittal (degrees)	Coronal (degrees)	Horizontal (degrees)
A	1.4	1.2	0.6
B	1.6	1.0	0.6
C	1.5	1.0	0.6

**Table 7:** Lumbar position variable error for all trials for all subjects by operator

Operator	Sagittal (range) (degrees)	Coronal (range) (degrees)	Horizontal (range) (degrees)
A	1.6 (0.3–3.8)	1.3 (0.3–3.4)	1.8 (0.2–3.6)
B	1.8 (0.4–4.2)	1.4 (0.1–4.4)	1.8 (0.4–3.6)
C	2.4 (0.0–5.1)	1.2 (0.4–3.3)	1.6 (0.3–3.8)

## Discussion

Two of the purposes of this study were to investigate the levels of agreement of independent palpation, and measurement of spinal motion between 3 separate operators using the modified CA 6000 spinal motion analysis system. Overall, the 'substantial' to 'almost perfect' inter-operator ICC levels achieved within this study are expected to facilitate future multi-center research. Previous work that had been carried out to ensure methodological precision [2,3] regarding palpation procedures, skin fixation pad placement, warm-up/familiarization movements and a standardized neutral starting position, were all part of the methodology of this multi-rater study, and may have contributed to the levels of agreement achieved by the operators in this particular investigation. In acknowledging some limitations within this present study, the results were necessarily generated with a convenience sample of volunteer subjects. The high level of agreement in palpation could also have been facilitated by the fact that all the subjects were within normal ranges for body mass index and most were either of mesomorphic or towards ectomorphic somatotyping. Spinal palpation, and therefore accuracy, can be less problematic with such subjects than with those of endomorphic body type or who are of higher body mass index. However, in order to minimize the potential risk of error, it is suggested that rigorous standardization of landmark location and palpation protocols, as well as data collection procedures, should be incorporated routinely into future inter-rater studies. The results obtained here suggest that the modified instrument may be an appropriate means to test future methodologies when relating palpation accuracy to ROMs achieved.

Within the available timescale, it was found impossible to blind each operator to the other operator's markings. The initial marks made by each operator to determine vertebral location were determined without visualization of the other operator's marks as the spine was palpated with the subject's back facing away from the operator. Each operator utilized his/her own color coded markings for placement of the OSI apparatus. This methodology was considered the most practical and also ensured that each operator placed the fixation pads for CA 6000 linkage attachment at the same place on each subsequent day of testing. Data from only three subjects indicated palpation differences among the three operators. The 2, 5, and 18 mm variability in palpation marker placement found between 2 operators in this study is similar to the variability of 10.9 to 17 mm differences between 3 pairs of manipulative physiotherapists in a study by Downey et al. [10]. Our findings were similar for the 18 mm and much less for the two other error measurements in this other study [10]. Windows representing the size of the particular spinous process were placed over the therapist's transcribed marks in the Downey et al. study [10]. A weighted kappa coefficient was used to determine the extent of agreement (0.92) between the pairs of therapists in Downey et al. [10], representing almost perfect agreement. As two of the error findings in our study were less than the Downey et al. [10] findings and the third was very similar, this present study suggests good inter-therapist segment palpation reliability. These differences seemed to have little effect on the resulting ROM measurements. Error is an important palpation consideration, but the results of this study suggest that when palpation is carried out by competent clinicians with good anatomical knowledge in combination with a validated instrument and standardized procedures for measurement, a minimal amount of specialized training between operators can be effective.

The third goal of this study was to examine the reliability of upright lumbar measures attained by each subject. The results are similar to those reported by Feipel et al. [11] utilizing the same OSI CA 6000 system, but with the standard strap fixation. Feipel et al. [11] also utilized a different testing procedure (sitting on a rocker board) with guidance given to each subject to a specific point in the range of motion. It might be inferred that a level of passive input from the operators in the Feipel et al. [11] study may have influenced subject positioning. By contrast, the subjects in this present study utilized upright standing posture, and were only given the command to attain or return to that upright standing posture without any other operator influence. Overall, our subjects were consistent in their attainment of the upright position of the lumbar spine with all 3 operators. The slight variation which occurred in the examiner palpation and placement of the hook bases at T12 had no apparent influence on these results.

## Conclusion

Our results suggest that palpation, measurement of spinal motion, and measures of upright lumbar spinal position, between different operators, can be consistent across subjects. Previous work to ensure methodological precision with the use of specific palpation techniques with the skin fixation pads using the modified CA 6000 spinal motion analysis system is expected to facilitate future multi-center research. The work is considered relevant to medical clinicians working in the fields of spinal dysfunction, surgical interventions, or occupational health. We suggest that any similar multi-center research should utilize a validated instrument and follow rigorous standardization of all aspects of the methodology employed, to ensure multi-rater reliability and repeatability throughout.

## Competing interests

The author(s) declare that they have no competing interests.

## Authors' contributions

All authors contributed equally to this work.

## Pre-publication history

The pre-publication history for this paper can be accessed here:



## References

[B1] Troke M, Moore AP (1995). The development of a new form of instrument fixation for the OSI CA 6000 spine motion analyzer. Man Ther.

[B2] Troke M, Moore AP, Cheek E (1996). Intra-operator and inter-operator reliability of the OSI CA 6000 Spine Motion Analyzer with a new skin fixation system. Man Ther.

[B3] Troke M, Moore AP, Cheek E (1998). Reliability of the OSI CA 6000 Spine Motion Analyzer with a new skin fixation system when used on the thoracic spine. Man Ther.

[B4] Troke M, Moore AP, Maillardet FJ, Cheek E, Wells NS, Sidhy PS (2001). Radiographic validation of measures recorded by the modified CA6000 spine motion analysis system. Rheumatology.

[B5] Troke M, Moore AP, Maillardet FJ, Hough A, Cheek E (2001). A new, comprehensive normative database of lumbar spine ranges of motion. Clin Rehabil.

[B6] Troke M, Moore AP, Maillardet FJ, Cheek E (2005). A normative database of lumbar spine ranges of motion. Man Ther.

[B7] Landis JR, Koch GG (1977). The measurement of observer agreement for categorical data. Biometrics.

[B8] Portney LG, Watkins MP (2000). Foundations of Clinical Research.

[B9] Schmidt RA (1988). Methodology for Studying Motor Behavior. Motor Control and Learning: A Behaviorial Emphasis.

[B10] Downey BJ, Taylor NF, Niere KR (1999). Manipulative physiotherapists can reliably palpate nominated lumbar spinal levels. Man Ther.

[B11] Feipel V, Parent C, Dugailly PM, Brassinne E, Salvia P, Rooze M (2003). Development of kinematic tests for the evaluation of lumbar proprioception and equilibration. Clin Biomech.

